# American Medical Association—Sixteenth Annual Convention

**Published:** 1865-07

**Authors:** 


### AMERICAN MEDICAL ASSOCIATION—SIXTEENTH ANNUAL CONVENTION.

We are indebted to the *Boston Medical and Surgical Journal* of June 15th for the following report of the meeting of the American Medical Association:

### PROCEEDINGS OF TUESDAY, THE FIRST DAY.

The American Medical Association began its sixteenth annual convention at the State House, Boston, in the Hall of the House of Representatives, on Tuesday, the 6th instant. A large number of delegates and members were present, occupying the entire floor of the hall, while the galleries were occupied by spectators. The officers of the convention were as follows:

*President*—N. S. Davis, M. D., of Illinois.
*Vice Presidents*—Wm. H. Murray, M. D., of Ohio; Worthington Hooker, M. D., of Connecticut; William Whelan, M. D., of District of Columbia; J. E. B. Heintze M D., of Maryland.
*Secretary*—William B Atkinson, M. D., of Philadelphia.
*Assistant Secretary*—Horatio R. Storer, M. D, of Boston.
*Treasurer*—Caspar Wister, M D., of Philadelphia.

At about half-past ten o'clock, the Convention was called to order, and prayer was offered by Rev. Dr. Lothrop, after which Dr. Henry J. Bigelow, of this city, delivered the address of welcome.

At the close of Dr. Bigelow's address, the roll of registered members was read by the Secretary.

The President then proceeded to deliver the annual address, and a copy of it was requested for publication.

The President read a communication from Mayor Lincoln, inviting the members of the Association to an excursion down the harbor of Boston to-morrow. It was unanimously voted to accept the invitation.

Papers and reports from special committees appointed or continued at the last session, being next in order, were called for, and the following were presented:

On Insanity—Report by Dr. H. R. Storer, of Boston. Referred to the Section on Practical Medicine and Obstetrics

On the Relations of Electricity to the Causes of Disease—Paper from Dr. Littell, of Pennsylvania. Referred to the Section on Practical Medicine and Obstetrics.

On Climatology and Epidemic Diseases of California—Reports by Drs. Logan of California, and B. H. Catlin, of Connecticut. Referred to the Section on Meteorology and Epidemics.

On Alcohol and its Relations to Man—Report by Dr. G. E. Morgan, of New York. Referred to the Section on Practical Medicine and Obstetrics.

On Autopsies and their Relations to Medical Jurisprudence—Report by Dr. T. C. Finnell, of New York. Referred to the Section on Medical Jurisprudence.

On the Introduction of Disease by Commerce, and the Means of its Prevention—Report by Dr A. N. Bell, of Brooklyn, N. Y. Referred to the Section embracing Hygiene.

On Exsections and their Relation to Conservative Surgery—Papers by Dr. Tewksbury, of Iowa, and Dr. Lyon. Referred to the Section on Surgery.

On Specialists and Specialties—The Committee were requested to report on Wednesday morning at 9 o'clock.

On the Rank of Medical Corps in the Army—Report by Dr. Tripler, U. S. A. Referred to the general session of Wednesday.

On the Rank of Medical Corps in the Navy—Report by Drs. Anderson, of New York, and others. Referred to the general session of Wednesday.

On Smallpox—Papers by Drs. Ramsey, of New York, and Nebinger, of Philadelphia. Referred to the Section on Hygiene.

Volunteer communications were then called for, and the following papers were presented:

On Ophthalmology—By Dr. Williams, of Cincinnati. Referred to the Section on Surgery.

Extraction of Foreign Bodies from the Ear and Nose—By Dr. Turnbull, of Philadelphia. Referred to the Section on Surgery.

On Staphyloraphy—By Dr. J. Mason Warren, of Boston. To be read at 3 o'clock P. M., on the 7th, at the Medical College.

On Surgery—By Dr. Henry J. Bigelow, of Boston. To be read at the Medical College at 4 P. M., of the 7th.

On the Functions of the Nerves of Sensation and Motion—By Dr. Haskell, of Rockport. Referred to the Section on Anatomy and Physiology.

On Dislocations of the Clavicle—By Dr. Holton. Referred to the Section on Surgery.

Dr. W. Marsden, President of the College of Physicians and Surgeons of Lower Canada, was introduced and made a brief address.

It was voted that a committee to nominate officers, consisting of one from each State, be appointed by the various delegations.

At fifteen minutes before two o'clock the Convention adjourned.

In the afternoon the several sections organized by the choice of chairmen and secretaries, and proceeded to the consideration of the reports and papers referred to them.

*Soiree and Promenade Concert at the Music Hall.*—In the evening the members of the Convention attended a soiree at the Music Hall, tendered to them by their professional brethren of Boston, and a few hours were most agreeably spent in social intercourse.

### PROCEEDINGS OF THE SECOND DAY.

The second day's session of the Association was opened at 8:20 A. M., by the President, Dr. N. S. Davis, of Chicago.

Permission was given the committee on nominations to retire for consultation at 9 o'clock. The roll of the additional delegates registered since yesterday's session of the Association was read by the Secretary. The number present was then announced to be 468.

Dr. Cox, of Maryland, offered a preamble and resolution, which were amended and adopted in the following form:

*Whereas*, Montrose A. Pallen, whose name appears upon the register as a permanent member of this Association, has been declared, under oath, before the military commission sitting at Washington at this date, to have been in complicity with an attempt to poison the Croton Reservoir, by which the city of New York is supplied with water—thus imperilling the lives of thousands of his fellow-citizens, therefore

*Resolved*, That the said Montrose A. Pallen has disgraced his manhood and the humane profession of which he is an unworthy member—that he is hereby indignantly expelled from this body—that the Secretary be required to strike his name from the roll of members.

Dr. Ordway, of Boston, subsequently presented a remonstrance, signed by about thirty members, against the action of the Association. Upon which a committee of three was appointed to prepare an answer to the paper. This committee made an elaborate report, sustaining the action of the Association.

It was voted that the final meeting of the Association shall begin on Friday morning at 9 o'clock.

A series of resolutions, offered by Dr. Garrish, of New York, of respect to the memory of the late Dr. Valentine Mott, were adopted by the Society.

On motion of Dr. Catlin, of Connecticut, a committee was appointed to prepare suitable resolutions on the death of Dr. Jonathan Knight, Dr. Benjamin Silliman, and Dr. Charles Hooker.

Reports and papers from special and other committees were next in order, and the following were presented:

On the Rank of Medical Corps in the Army—Report by Dr. Tripler, U. S. A. Accepted and adopted. On motion of Dr. Cox, of Maryland, a resolution was adopted that the committee be continued, and that each member of this Association use his personal effort with members of Congress to secure a more elevated grade for the entire medical corps in the Army.

On motion, it was voted that the report of the committee and the subject discussed therein be presented to the next Congress by the President of this Association.

On the Rank of Medical Corps in the Navy—Report by Dr. James Anderson, of New York, read by the Secretary. Accepted and adopted.

On motion, it was resolved that the committee be continued, and that the same course be taken with this report by the President as was voted to be taken with the report from the medical corps in the Army.

The report of the Committee on Publication was submitted by Dr. F. G. Smith. Appended to the report was a resolution authorizing the committee to invite each member to contribute such additional sum as will enable them to defray the expense of publishing the forthcoming report, provided the amount in the hands of the treasurer shall prove to be insufficent for the purpose.

The report of the Committee on Prize Essays was submitted by Dr. D. H. Storer, of Boston. It was announced that a prize had been awarded to the author of the dissertation on the "Surgical Treatment of Morbid Growths in the Larynx, bearing the motto "*quod vidi scripsi*." On opening the envelope bearing the corresponding inscription, the author was announced to be Dr. Louis Elsberg, of New York.

The premium offered at the last annual meeting for "the best short and comprehensive tract calculated for circulation among females," on Criminal Abortions, was awarded to Dr. H. R Storer, of Boston.

The Report on Medical Education was submitted by Dr. Antisell. Referred to the Committee on Publication.

The Report of the Treasurer was submitted, showing the amount in the treasury to be $301.95.

A paper on "Compulsory Vaccination" was referred to the Committee on Medical Jurisprudence.

The report of the Committee on Revision of the Constitution was submitted by the President of the Association, Dr. Parsons occupying the chair temporarily. The report was accepted and adopted, and the thanks of the Association were voted to the Committee for the satisfactory manner in which they had discharged their duty.

The report of the Committee on Necrology was submitted without reading, and referred to the Committee on Publication.

The Nominating Committee reported a resolution that the several sections shall select the subjects germain to their organizations, and also appoint the committees thereon.

Dr. Toner, of Washington, offered an amendment to the Constitution, that delegates on registering their names shall pay the sum of $5.00, and permanent members, $3.00 The amendment, according to the Constitution, lies over until next year.

The Committee on Nominations, through their chairman, reported that they had decided upon Cincinnati, Ohio, as the next place of meeting of the Association. Dr. Cox, of Maryland, offered an amendment, substituting Baltimore, Md., for Cincinnati, Ohio. A spirited debate followed, and at length the motion to amend was carried, and Baltimore decided upon as the next place of meeting.

A committee was appointed to draw up a series of resolutions on the death of Dr. S. D. Willard, late Secretary of the New York State Medical Society, and also to prepare a short biographical sketch of his life, both to be submitted to the Committee on Necrology.

The Association adjourned at 2 o'clock.

PROCEEDINGS OF THE THIRD DAY.

The Convention was called to order at 8¼ A. M., by the President.

On motion of Dr. Burns, of Pennsylvania, a resolution was adopted requesting the sections or committees to which papers had been referred, to use special judgment and discretion in assigning such papers to the sections to which they properly belong, and in selecting such as they may consider worthy of publication.

On motion of Dr. Mayburry, of Pennsylvania, it was

*Resolved*, That the Permanent Secretary be instructed to prefix to the List of Officers and Permanent Members, a list of Ex-Presidents and Ex-Vice Presidents of the Association.

Dr. Jarvis, of Dorchester, inquired concerning the functions of the Committee on Publication. A discussion on this question followed, in which the President, Drs. Jarvis, Sayre of New York, Tripler and Hillard participated. By a vote of 45 to 37, a resolution was adopted giving the committee discriminating power.

On motion of Dr. Jarvis, of Dorchester, it was voted to establish a section on Psychology.

Dr. Garrish, of New York, moved that a section on Ophthalmic Medicine be established.

Dr. Twitchell, of New Hampshire, favored it. Dr. Williams, of Cincinnati, also favored the establishment of such a section, as there was need for thorough investigation of a science which had in the past ten years become, as it were, new, all the old landmarks having been swept away. The motion was opposed by Dr. Burns, of Pennsylvania, Dr. A. H. Stevens, of New York, and others. It was finally rejected.

On motion of Dr. Hibberd, of Indiana. the section on Anatomy and Physiology was abolished, the subject of Anatomy being assigned to the surgical section, and that of Physiology to the section on hygiene.

A resolution was offered by Dr. Couper, of Delaware, and read by Dr. Tripler, of the United States Army, that this Society send a delegate annually to the convention of medical superintendents of insane establishments, and that efforts be made to cause a more intimate relation between the two organizations; the delegate to be selected by the President. Amendments to the Constitution, to be acted upon at the next annual meeting, were offered in relation to permanent members, delegates from local societies, and members by invitation.

The Committee on Nomination reported that the time of the next meeting shall be in June, and submitted the following list of officers:

*President*, Dr. D. H. Storer, of Massachusetts; *Vice-Presidents*, Drs. James F. Hibberd, of Indiana; S. O. Almy, of Ohio; T. C. Dunn, of Rhode Island; W. P. Johnson, of the District of Columbia; *Assistant Secretary*, Dr. J. E. Morgan, of Maryland.

*Committee on Arrangements*, Dr. C C. Cox, W. C. Van Bibler, Franklin Donelson, L. H. Steiner, George G Miltenberger, William Whitridge, J. E. Morgan, all of Baltimore.

*Committee on Publication*, Drs. F. G. Smith, of Pennsylvania; H. F. Askew, of Delaware; William Mayburry, of Pennsylvania; W. B. Atkinson, of Pennsylvania; H. R. Storer. of Massachusetts; Caspar Wister, of Pennsylvania.

*Committee on Prize Essays*, Drs. A istin Flint, senior, James R. Wood, Ellsworth Elliot, E. Krackowitzer, D. C. Enos, all of New York.

*Committee on Medical Education*, D s. S D Cross, of Pennsylvania; G. P. Twitchell, of New Hampshire; C. A. Pope, of Missouri; O. W. Holmes, of Massachusetts; Graf on Tyler, of D strict of Columbia.

Medical Literature (present committee continued over), Dr. Charles A. Lee, of New York; T. F. Rochester, of New York; C. C. Cox, of Maryland; Albert Smith. of New Hampshire; A. Nebinger, of Pennsylvania.

*Committee on American Necrology*, Drs C C. Cox, of Maryland; E. B. Stevens. of Ohio; W. F. Peck of Iowa; H. Van Dusen, of Wisconsin; Noble Young. of the District of Columbia; Josiah Simpson. of the United States Army; J. C Weston, of Maine; Henry Bronson. of Connecticut; Henry Noble, of Illinois; Charles Eversfield, of the United States Navy; William B. Fletcher, of Indiana; I. C Hupp, of Western Virginia; J Mauran, of Rhode Island; William K. Bowling, of Tennessee; J. P. Fitch, of New Hampshire; James Couper, of Delaware; W. L. Linton, of Missouri; Chas. L. Allen, of Vermont; H. G. Clark, of Massachusetts; J. H. Griscom, of New York; E. M. Moore, of New York; Charles A. Logan, of Kansas; William B. Little, of California; Stuart, of Minnesota; Henry Miller, of Kentucky; S. G. Armor, of Michigan; William Pierson, of New York; Fl ming, of Pennsylvania; E. Wallace, of Pennsylvania.

The report was adopted as a whole, with the exception of the time of holding the next annual meeting. It was voted to meet on the first Tuesday in May, 1866, instead of June.

Dr. Hooker, of Connecticut, from a special committee, reported the following resolutions on the death of Dr. Knight, Dr. Charles Hooker and Dr. Benjamin Silliman, all of Connecticut. The report was accepted and the resolutions adopted.

*Resolved*, That in the death of Dr. Jonathan Knight, twice a President of this Association, the duties of which office he discharged with extraordinary ability, the Medical Profession has lost one of its brightest ornaments; an eminent Surgeon who was conservative in the true sense of that term; a teacher, who by his transparent clearness and terse eloquence, unsurpassed among medical men in this country, exerted through fifty years a wide influence upon the character of the American medical mind; a Christian gentleman, whose genial qualities won for him, to an extent seldom witnessed, the affections not only of intimate friends, but of all those who knew him

*Resolved*, That in lamenting the sudden departure from this life of Dr. Charles Hooker, at the height of his usefulness, we cherish the memory of one whose independent mind, restless activity of Christian faithfulness obtained for him great eminence, both as a physician and a teacher of medicine; and we call to mind with special pleasure his earnest devotion from the outset to the interests of this Association.

*Resolved*, That we revere the memory of Professor Benjamin Silliman, who, as a leader in the diffusion of science in this country, and in other countries also, was one of the great benefactors of the race, and who by his urbanity, kindness of heart, and cheerful piety, enhanced to an uncommon degree his influence upon the community as a man of science.

Dr. Furman, of New York, presented resolutions adopted by the State Medical Society of New York, and referred to the National Medical Society, against newspaper advertising, except merely the card of the physician. The resolutions were laid on the table, to be taken up after the reading of the report of the committee on specialties and specialists, which was specially assigned for this time.

Dr. Julius Homberger, of New York, chairman of the committee on specialties and specialists, presented an elaborate individual report favoring advertising by specialists and opposing the same course by general practitioners. The report was laid upon the table for the present, and a second report was submitted by Dr. H. R. Storer, of Boston, also a member of the committee, defending specialists, placing them in a high position. He presented strong arguments concerning the benefits and advantages of special investigations both to practitioner and patient.

Dr. D. Humphreys Storer spoke concerning a passage in the report relating to himself, denying that he was ever a specialist, though he did not object to any person being a specialist. Dr. H. R. Storer, in reply, defended his own ground. The report was laid upon the table temporarily.

Dr. W. Hooker, of Connecticut, another member of the committee, reported that he had not had sufficient time to investigate the report of Dr. Homberger, and therefore could not give an opinion concerning it. With the report of Dr. H. R. Storer he could not entirely agree, especially in that part of the report which referred to specialists as the leaders of the profession.

The reports were then taken from the table for consideration.

Dr. Martin made a motion that the reports be referred back to the committee for consideration another year, and supported his motion at some length.

Dr. Bissell, of New York, moved to amend the previous motion, and moved that the reports be referred to the committee on medical ethics.

Dr. Toner, of Washington, criticized the reports in a severe manner.

Dr. Homberger, of New York, defended his position, and stated as the reason why he did not send his report to the other members of the committee at an earlier date, that he knew they would not agree with him and would not sign it. His remarks were not received very favorably by the members.

Dr. Mayburry, of Pennsylvania, favored strongly the referring of the reports to the committee on medical ethics.

The question was then taken on referring the reports to the committee on ethics, and decided in the affirmative. On motion of Dr. Twitchell, of New Hampshire, the committee on ethics was instructed to report some definite action on this subject.

The Convention adjourned at half past twelve o'clock.

The afternoon was devoted to an excursion down the Harbor to Deer Island and Fort Warren. The company disembarked on Long Island, where a collation was served; after which speeches, by His Honor the Mayor and members of the Association, were made.

### PROCEEDINGS OF THE FOURTH DAY.

The Association was called to order at nine o'clock by the President. Reports from sections were received and appropriately referred, and it was voted that such bills as might be presented therein should be paid by the treasurer of the Association. After the transaction of some unimportant business, Drs. Leidy, F. G. Smith and H. Hartshorn were appointed a committee on the paper of Dr. Colt, on "the microscope."

At this point, His Excellency the Governor of the Commonwealth, preceded by the Sergeant-at-Arms, and accompanied by Assistant Surgeon General Hooker, entered the hall, and was received by the Association, the members rising in their seats.

A special committee on insanity was appointed, consisting of Drs. Alfred Hitchcock, of Massachusetts; Isaac Ray, of Rhode Island; S. H. Tewksbury, of Maine; B. F. Barker, of New York; and J. S. Butler, of Connecticut.

The Secretary read the following resolution offered by Dr. Garrish, of New York:

*Resolved*, That the American Medical Association, with heartfelt and national pride, extend to the Army and Navy Surgeons their thanks for the prompt manner in which they have met the calls and perils of the battlefield, demanded by our country.

*Resolved*, That we tender to the families and friends of those who have sacrificed their lives in the cause of humanity, our earnest sympathies for the bereavement they have sustained. They have restored to us and the whole nation a peaceful and happy home for all future time.

The resolutions were adopted.

On motion, the duty of revising the list of permanent members for republication was referred to the committee on Medical Necrology.

Dr. Burns, of Pennsylvania, presented the following resolutions, which were unanimously adopted:

*Resolved*, That the American Medical Association, in closing their annual session in the city of Boston, June 9th, 1865, do most sincerely tender a vote of thanks to their medical brethren, the Governor of Massachusetts, city authorities and inhabitants of Boston, for their very kind and generous hospitalities to the assembled delegates from all parts of our country; and in bidding them farewell we shall ever cherish the remembrance of their kindness, and express the hope that prosperity, with every domestic, social, intellectual and religious blessing, may be their abiding portion.

By Dr. Kennedy, of New York, resolutions were offered as follows:

*Resolved*, That the thanks of the American Medical Association be and are hereby tendered to His Honor Mayor Lincoln and the authorities of the city of Boston for the kind reception and splendid entertainment given to the Association during their stay in the city.

*Resolved*, That the thanks of the Association are due, and are hereby presented, to the committee of arrangements of the Association for the faithful and satisfactory manner in which they have discharged the arduous duties imposed upon them.

*Resolved*, That the thanks of the Association be presented to our President, Dr. N. S. Davis, for the singularly able and impartial manner in which he has discharged the duties of his office.

The following was offered by Dr. VanKleek, of New York:

*Resolved*, That this is a National Association, and as, in its earlier days, we enjoyed with pleasure and profit the intercourse of our professional brothers of all parts of our country, and as the unhappy feud which for four years divided the nation, has now ceased and peace has again come, we trust forever,—so we hope soon again to meet our members and delegates from the South on this platform of fraternization, and to this end we extend to them an earnest invitation and promise them a cordial welcome.

The following by Dr. Bowditch was offered by Dr. Hibberd:

*Resolved*, That this Association has learned with deep regret that there are fears of famine, and consequent disease and pestilence, occurring in some parts of Georgia and South Carolina, passed over by Gen. Sherman's army in its victorious course.

*Resolved*, That this Association would urge upon the Sanitary and Christian Commissions to send agents to learn the exact facts, and, if need be, to take prompt and efficient measures to prevent or mitigate such a distressing result.

The following was offered by Dr. Bronson:

*Resolved*, That the thanks of this Association be and they are hereby tendered to Major John Morrisey, the Sergeant-at-Arms of the Massachusetts Legislature, and his assistants, for the very able and efficient manner in which they have performed the functions of their offices during the sessions of the Association.

The resolutions were all unanimously adopted.

At a quarter past one o'clock, the Convention adjourned to meet on the first Tuesday in May, 1866, in the city of Baltimore.

The annual meeting of the Association of 1865 has been more largely attended than on any previous occasion, six hundred and sixteen members and delegates having registered their names on the books of the committee. It is generally regarded as having been a most interesting and successful Convention.

